# Occupational Factors Affecting Women Workers’ Sexual and Reproductive Health Outcomes in Oil, Gas, and Mining Industry: A Scoping Review

**DOI:** 10.3389/phrs.2022.1604653

**Published:** 2022-04-28

**Authors:** Rina Hariniaina Razafimahefa, Jerico Franciscus Pardosi, Adem Sav

**Affiliations:** ^1^ School of Public Health and Social Work, Faculty of Health, Queensland University of Technology, Brisbane, QLD, Australia; ^2^ Centre for Accident Research and Road Safety, Queensland University of Technology, Kelvin Grove, QLD, Australia; ^3^ School of Psychology and Counselling, Faculty of Health, Queensland University of Technology, Brisbane, QLD, Australia

**Keywords:** women, women health, sexual and reproductive health, work and occupation, oil and natural gas, mining industry

## Abstract

**Objectives:** Globally, female workers workforce in Oil, Gas, and Mining (OGM) industry have increased significantly. The complexities of the OGM operations and the extensive exposure to workplace hazards potentially affect the health status of workers, including sexual and reproductive health (SRH) outcomes of female workers. Yet, the current state of knowledge on SRH issues in OGM contexts seems to be limited and fragmented. This scoping review aims to identify the occupational factors that influence women’s SRH outcomes in OGM industry.

**Methods:** This scoping review followed the Joanna Briggs Institute’s guidelines (PRISMA) and was conducted in five databases, including the citation chaining *via* Google Scholar and manual search through relevant organisations and Government websites. Sixteen articles met the inclusion criteria and were analysed.

**Results:** Despite the scarcity of evidence, chemical and physical are found to be the predominant factors greatly influencing women workers’ SRH outcomes in OGM. Most studies showed menstrual and cycle disorders, and risky pregnancy as key SRH issues. However, menstruation disorder was considerably linked with psychological and organisational factors.

**Conclusion:** This review suggests further empirical research on the relationship between OGM occupational hazards and women workers’ SRH. This will contribute to improvements in workplace safety legislations, measures, policies, and management systems taking into account women’s needs.

## Introduction

The oil, gas, and mining (OGM) industry is one of most hazardous for workers. This is due to the complexity of the processes, workers’ exposure to dangerous substances, and work-related accidents [1, 2]. Despite its high-risk nature and heavy focus of males, there has been a worldwide significant increase in female workers in OGM industry [3]. For example, women represented 38% of newly hired professionals in the Exxon Mobile, and American multinational oil and gas corporations, and 31% in Shell (a British-Dutch multinational oil and gas company) in 2007 [4]. In Australia’s OGM industries, women participation has increased from 13.3% to 14.3% between 2009 and 2016, from 13.5% to 14.7% between 2011 and 2017 in European Union [3, 5]. The most significant increase has been in South Africa, from 3% to 20% between 2002 and 2019 [6].

Many women choose to work in OGM industry because of the employment opportunities, potential career advancement, higher incomes, and family health insurance [7,8]. In return, women’s participation in OGM industry has been acknowledged as a powerful contributor of companies’ economic growth and sustainable development [7, 9–13]. Research suggests because of female workers’ leadership skills and higher organisational performance in OGM industries, there has been an increase in family-friendly practices, workers’ productivity, and a reduction in both turnover and workplace risks [13–15].

### Oil, Gas, and Mining Main Operations and Occupational Hazards

In OGM industry, operations are mostly undertaken under the fly-in/fly-out/drive-in/drive-out (FIFO/DIDO) system [16–18]. This practice requires long distance travel by plane or vehicle to work in remote areas during fixed period, without families. Workers are provided accommodation, food, and return travel during a limited number of days [19]. Employees work under pressure, with an inflexible work schedule, uncomfortable postures, and in noisy, hot, or cold environments, requiring strenuous physical and mental resilience [20–22]. These conditions indicate the potential exposure of OGM workers to chemical, physical, ergonomic, biological, and psychological hazards [16–18, 23–25]. As a result of exposure to such hazards, workers in OGM industry are at risk of experiencing diseases, injuries, disabilities, or fatalities. Women workers in particular are also at risk of sexual and reproductive impairment [16–18, 21, 23].

### Women’s Sexual and Reproductive Health Status

Sexual and reproductive health is defined as “the state of physical, emotional, mental, and social wellbeing concerning all aspects of sexuality and reproduction, as well as concerning disease, dysfunction and infirmity” [26]. It contributes to the individual quality of life and community’s sustainable development [27]. Women’s reproductive system is complex, with specific gene function exhibiting hormonal cyclic changes to ensure fertilisation and pregnancy [28–30]. Maternal deaths, poor pregnancy outcomes, and sexual health problems, such as HIV/AIDS, still remain as global SRH key challenges, especially among women in low- and middle-income countries (LMICs) [31]. It is estimated that there are approximately 810 daily maternal deaths in 2017, 2 million stillbirth cases and 295,000 newborn deaths within 28 days of birth every year [32–34]. Stillbirth and neonatal deaths might occur due to sexually transmitted diseases (STDs) during gestational phase, such as congenital syphilis, human papillomavirus infection (HIV), chlamydia, gonorrhoea and trichomoniasis [26, 34–36]. Finally, women can also experience other forms of diseases during pregnancy and postnatal stages, including diabetes, hypertension, toxoplasmosis, rubella, urinary tract infections, obesity, and mental disorder [26, 37].

### Physical Affects of Maternal Exposure to Oil, Gas, and Mining Hazards

Several studies indicate that maternal exposure to OGM hazards affects women’s physical and/or mental health, pre-conception germ cells and hormones, and foetal development [38–41]. In fact, there have been limited but concerning reports that women employed in OGM industry may experience abnormal menstruation, congenital malformation, pregnancy complications, miscarriage, stillbirth, preterm labour, low birth weight, birth defects and other gynaecological inflammation and hyperplasia [23, 41–46]. For example, research in Nigeria, Kenya, Louisiana, Ecuador, Colorado, China, Iraq, indicate the manifestation of maternal hypertensive disorders, depression, gestational diabetes mellitus, congenital anomalies associated with oil pollution and gas flaring [47–49]. Also, HIV and STD infections were identified among female miners, health care workers and communities in Latina America and the Caribbean [50]. This was mainly because of the oversupply of alcohol and drugs and sex services due to mineworkers’ high income, migration, accommodation system, family distance and communities’ poverty [50]. Female miners can be infected by having an unprotected sex with infected partner or any person working in the OGM industries or from the community.

Although numerous studies have drawn attention to the relationship between women’s work in OGM industry and their sexual and reproductive health, the current state of knowledge seems to be limited and fragmented [40–43, 51, 52]. For example, most of the literature has appeared to focus on the chemical hazards. Although chemical hazards indeed pose an important risk to women’s sexual health and reproduction, little information is available on the other categories of hazards, such as physical, biological, etc. Furthermore, focusing on chemical hazards only may limit the application of current interventions to women’s sexual health and reproduction issues due to the wide variety in OGM workplaces in terms of technology, work processes and organisations [26, 40, 41, 43, 51–53]. Hence, the crucial first stage in understanding the how the OGM industry affect women’s sexual and reproductive health is to scope the body of literature. This will assist health professionals and policy makers with developing the most efficient workplace health and safety strategies to reduce the risk of sexual and reproductive health issues among working women in such a high-risk industry.

Finally, a review of female workers’ sexual and reproductive health issues in OGM industries is timely given that 70% of female workers in OGM industry are 20–54 years old globally [54–56]. This suggests that most women working in OGM industry are within childbearing age period. Nevertheless, considering the decline of women’s fertility after the age of 32, their pregnancy plans and reproductive health status could be threatened [57–59]. Women’s participation in the workforce Employment and their decisions to delay pregnancy are argued to be among the causes of this SRH issue [27, 60–64].

The aim of this study is to review the current evidence to understand the occupational factors affecting sexual and reproductive health status among female workers in OGM industry. Although we use a systematic process to search for literature, our aim is not to assess the quality of this body of literature, which is conducted in a systematic review. Instead, we map the literature, and identify the gaps, and incite future research for a scientific-based knowledge about this field and a gendered-based health and safety management system, policies, and practices.

## Methods

This scoping review, which was guided by the principles established by Arksey and O’Malley, was conducted from July to August 2020 [65]. We followed Joanna Briggs Institute’s Preferred Reporting Items for Systematic reviews and Meta-Analyses extension for Scoping Reviews (PRISMA_ScR checklist) as shown in Supplementary Appendix S1 [66, 67]. The searches of articles from 2000 to 2019 were undertaken in five relevant databases, including CINAHL, Embase, PubMed, Scopus, Web of Science as shown in Supplementary Appendix S2. The search terms focused on Population, Exposure and Outcomes elements as presented in Table 1.

**TABLE 1:** Search terms used for the searches (Queensland, Australia. 2020).

(woman OR women OR Female* OR maternal) AND (Worker* OR workforce) AND (Oil* OR Gas* OR Oil and Gas* OR “oil industr*” OR “Gas industr*” OR “Oil and gas industr*” OR Mining OR Mine* OR fracking OR “hydraulic fracturing” OR petrochemical OR “Petrochemical plant*”) AND (“reproductive health” OR “sexual health” OR “sexual and reproductive health” OR “reproductive disorder*” OR pregnan* OR contraception OR “sexually transmitted disease” OR childbirth OR childbearing OR abortion OR “spontaneous abortion” OR antenatal OR fertility OR infertility OR sterility OR subfertility OR “preterm birth” OR “birth defect*” OR “congenital malformation” OR “congenital disorder*” OR “menstrual cycle” OR “ovary insufficiency”)

Additional citation chaining in Google Scholar, hand searching and browsing organisations and government websites were performed [1, 3, 33, 34, 68, 69]. This is mainly to generate more relevant articles and avoid missing significant evidence that would provide more comprehensive findings to the review. During the literature search, the authors also received assistance from a librarian.

All studies were exported into Endnote X9 citation management software [70] and screened for eligibility based on the following inclusion criteria: articles investigating female workers, sexual and reproductive issues, occupational hazards, large-scale OGM industry considering other associated operations, such as manufacturing and transport, primary data. Primary data included qualitative, quantitative, mixed method, textual papers, reviews, and grey literature, published and unpublished studies. Studies: published prior to year 2000, without full-text, in non-English languages, based on animal experiments, involving solely male workers or female community populations, focussed on non-occupational factors or non SRH diseases, safety hazards and risks, policies, interventions or treatment investigations, in industries other than OGM, small-scale mining, and OGM marketing activity were excluded. Female community was excluded due to the study focus on workers and their low exposure to the mining activities. Additionally, as the review was operational in focus, studies that concentrated on commercial and marketing activities in OGM were excluded.

Included studies were extracted by the first author using the inclusion criteria. The extracted studies were sorted in a charting table (Table 1), which included information on the author(s), year of publication, countries, objectives, study design, sample size, OGM types, occupational factors, sexual and reproductive status outcomes, and summary of the key findings. The results were thematically analysed and collated within six key themes of occupational factors.

## Results

**FIGURE 1:**
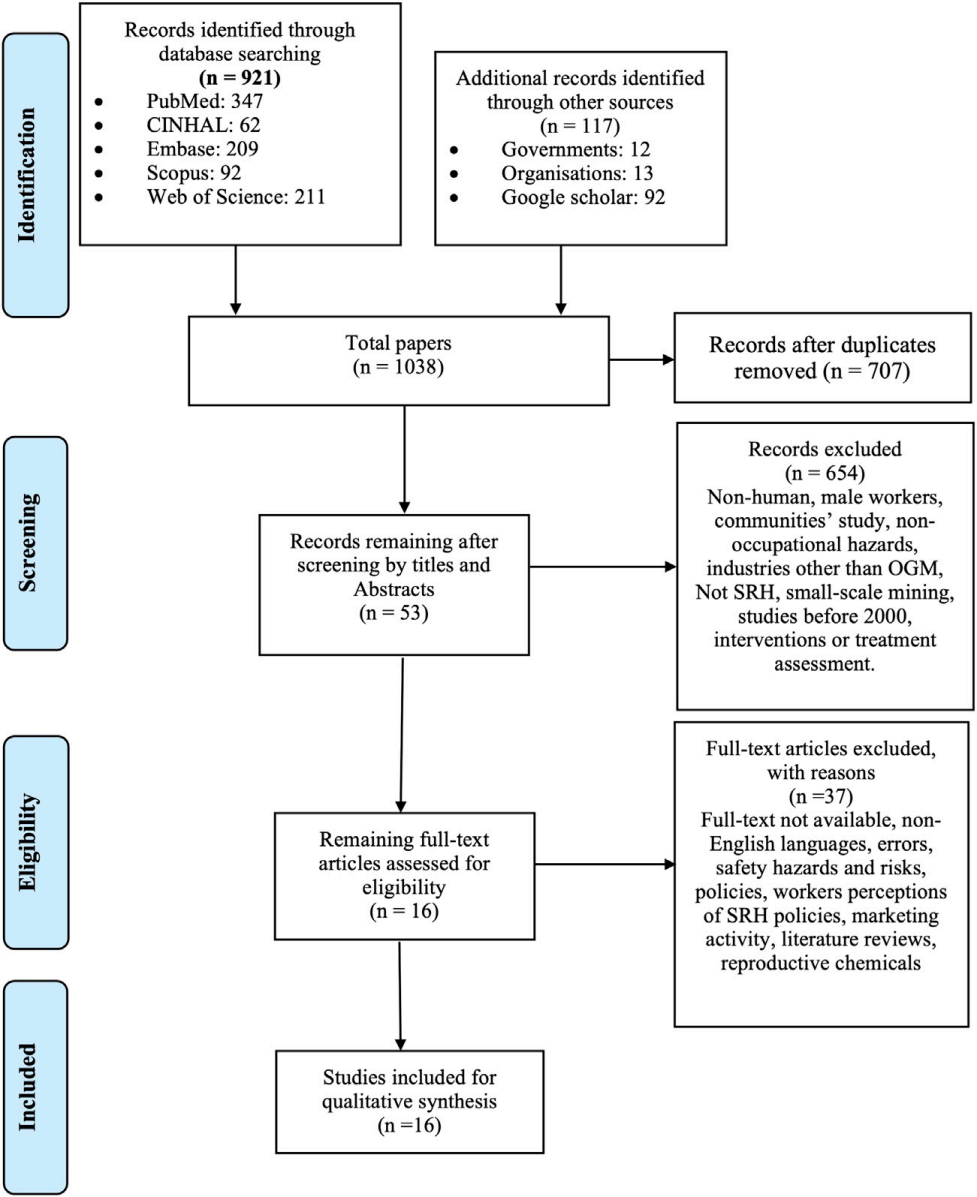
PRISMA flow diagram (Queensland, Australia. 2020).

The scoping review initially identified 1,038 studies (Figure 1) and sixteen were included in the final analysis (see Table 2).

**TABLE 2:** Data extraction of included studies (Queensland, Australia. 2020).

References	Aim/purpose	Countries	Study design	Sample size	OGM	Occupational factors specific findings	SRH outcomes	Main findings
[79]	Determine the experience of women in the platinum mining industry in South Africa and the impacts of working conditions, harassment, physiological and physical conditions, ergonomics, discrimination	South Africa	Qualitative Literature study, empirical investigation	14 (31–35 years old)	Mining	Physical: physical strength, physiological aspect, heat, cold, noise, dust, ventilation pressure. Organisational: discrimination, management negligence, male worker dominance and disrespect, work-life balance, long working hours, night shift, perception of pregnancy, inappropriate change room and sanitary facilities Psychological: stress and constant fear of harassment, men injure, injuries Ergonomic: working in dark, damp condition, small space, heavy machine, female physiology Chemical: dust, gasses	Prenatal: Menstruation and cycle disorder Pregnancy: Risky pregnancy Violence: Sexual harassment (verbal and physical)	Verbal and physical sexual abuse causing stress, fear and insecurity feeling Pregnant black women workers complain about mistreatment and negligence High ambient temperature, low humidity, thermo-regulation of female underground miners causing painful menstruation periods
[86]	Determine HIV prevalence among employees, mostly male populations	South Africa, Botswana, Zambia	Quantitative	44000 (>20 years old)	Mining	Not applicable	Sexual Health: HIV/AIDS	1 in 5 positive cases of HIV/AIDS. Females HIV prevalence: 20–39 years: 14.6% and 14.9% 40–49 years: reduction to 4.9% *Females prevalence higher than males between 20 and 29 years (14.6%/ 13.7%). *Males prevalence higher than females between 30 and 39 years (23.1%/ 14.9%) and 40–49 years (12.4%/ 4.5%)
[84]	Describe mining occupational health and safety conditions, issues, and new actions	South Africa	Transaction paper (Psychological wellbeing of women operating mining machinery)	N/A	Mining	Physical: radiation, heavy physical work, equipment design for men, heat and vibration, noise Chemical: inorganic solvents, air pollutants, toxic metals, Silica, and coal dust. Biological: No details. Ergonomic: No details	Sexual Health: HIV/AIDS Reproductive problems (general)	Male sexual harassment, intimidation or assault causing fear and frustration. Exclusion of married women with family burden, weak or unhealthy in the recruitment process. Appearance of uncommon diseases including AIDS among female workers and communities transmitted from male labourers
[85]	Assess occupational health and safety risks of Mine and heavy industries and provide a safe system of work to women of reproductive age, their unborn children and all working mothers who are breastfeeding	South Africa	Conference paper (The Southern African Institute of Mining and Metallurgy Hard Rock Safe Safety Conference 2009)	N/A	Mining	Physical: vibration, extreme heat, noise, radiation, work in compressed air, physical strain Psychological: stress and anxiety Ergonomic: physical and mental strain, prolonged sitting and standing Chemical: Carbone monoxide, Ethylene oxide, lead, Polychlorinated Byphenyls, organic solvents, pesticides, alcohol, tobacco smoke Biological: Cytomegalovirus, Hepatitis, HIV, rubella varicella, toxoplasma gondii	Prenatal: Menstruation and cycle disorder Pregnancy: embryo toxicity, risky pregnancy, low fetal blood supply, detrimental fetus, congenital malformation; miscarriage, stillbirth Delivery: Premature labour Postnatal: child abnormal development, physical and mental abnormalities Sexual health: HIV/AIDS Violence: Sexual harassment	Childbearing female workers SRH exposure to occupational hazards. Physical and psychological limited capabilities. SRH: menstruation and cycle disorders, genetic mutation abnormalities, infertility, embryo toxicity, congenital malformation, retarded growth and development defects, stillbirth, miscarriage, deep vein thrombosis, varicose veins, premature labour, birth defects, AIDS, and other communicable diseases)
[71]	Defend women as employees in the mining industry to ensure that work is undertaken in a safe and healthy way to protect their well-being at work	South Africa	Quantitative Inaugural lecture based on South Africa DMS cross-sectional survey in 2009	N/A	Mining	Psychological: acute and chronic stress due to discrimination; work-life imbalance Chemical: Reproductive toxicants Ergonomic: Heavy tools and equipment, male-designed Personal Protective Equipment (PPE), heavy material handling and lifting Physical: heat stress, noise, dust, heat, radiation	Prenatal: Hormonal imbalance; infertility Pregnancy: congenital malformation, miscarriage, Risky pregnancy Postnatal: birth defects	Change in hormone levels and fluid balance due to occupational stress. Reduction of fertility and impacts on fetus and newborn by exposure to toxic pollutants and improper size of PPE (3.6% of miscarriage and 1.3% of pregnancy complication).Urinary tract infection caused by inadequate sanitary facilities
[80]	Explore female machine operator’s experience of being a female blue-collar employee in the mining industry and FIFO practice	Australia	Qualitative Interview/ open-ended questions Interpretative Phenomenological Analysis	19 (22–46 years old)	Mining	Organizational: FIFO system, work schedule, Relationship with co-workers, work and family balance	Prenatal: Subfertility and motherhood rejection	Gender discrimination. Limited family life and relationship due to FIFO system. Hesitation to building family or decision to postpone pregnancy to the perceived difficulties of balancing work and parenthood
[82]	Determine the perceptions regarding women’s health and safety working in core mining positions, their challenges	South Africa	Mixed Structured questionnaire Individual and group interviews	156 (Age not given)	Mining	Organizational: legislations and, code of good practice, guidelines, and programs. Physical: strenuous physical efforts vibration, heat, eight hours work women physical capabilities, tools, and equipment	Prenatal: Menstruation and cycle disorder Pregnancy: Risky pregnancy	Lack of organisation’s pregnancy policy. Tiredness, back pain, and painful periods linked with work physical demand, use of male-designed tools and PPE, exposure to different physical hazards such as vibration, heat, eight hours
[72]	Examine non-communicable disease-infectious disease overlap in the specific comorbidity rates for key diseases in an occupational cohort in Papua, Indonesia	Papua, Indonesia	Quantitative Diagnosis and data collection	21513 men; 1035 women (18–68 years old)	Mining	Not applicable	Sexual Health: HIV/AIDS	HIV and AIDS infection did not manifest simultaneously with the other non-communicable diseases. However, its presence is notable in number of people with malaria (14.4%) and tuberculosis (6%) cases
[83]	Determine how families and relationships affect the career decisions, progression and outcomes of technical professional women in the Australian mining industry?	Australia	Mixed Statistical analysis of survey data analysis Life course timelines/face to face interviews. Semi-structured face to face interview	686 (30–63 years old)	Mining	Organizational FIFO system, inflexible time, work schedule	Prenatal Subfertility and motherhood rejection	Work-life imbalance due to FIFO/DIDO” system. Female workers between 25 and 44 years with young children and in lower positions are more concerned. Lower rate of married women (37% against 74%) and mothers (44% vs. 74%) in Mining compared to men
[81]	Observe and investigate women in Mining experience as caregivers and being exposed to highly contagious and resistant diseases that adversely affect their day-to-day lives and family relations	South Africa	Qualitative Field observation, survey, formal and informal interview, the researcher concluded thoughts	13 women	Mining	Organizational: Company pregnancy policy, management value on women SRH rights, discrimination, PPE provision, Infrastructure facility, Physical: Strenuous activities, heavy machinery operations, vibration, extreme heat	Pregnancy: Risky pregnancy	Disharmony of safe pregnancy and secured employment due to the lack of women SRH rights in Mining Industries policy and management. Thus, pregnancy is hidden until higher gestational age. Mental and physical strains threatening the mothers and the fetus health conditions
[73]	Investigate the association between female reproductive status and risk of spontaneous abortion among female workers in the Jinchang Cohort	Jinchang, China	Quantitative Survey prospective cohort study	18,834 (34–60 years old) 4 men (23–45 years old)	Mining	Physical: Fatigue. Psychological: Strong stress, anxiety, and pressure, emotional change Female reproductive status: the advanced age of the mother, last pregnancy, previous spontaneous abortion, low pregnancy weight, high fertility age, number of pregnancies, uterine cavity-related infection, difficulties to implement embryo	Pregnancy: Spontaneous abortion (<28 gestational weeks). Postnatal: Birth weight	41.06% of Spontaneous abortion due to fatigue. Other influencers: (a) Occupational stress, anxiety, and emotional change (b) Number of pregnancies, mother age, reproductive status, previous miscarriage, operation, or uterine cavity-related infection. 33.28% cases of spontaneous abortion in Petrochemical company. Higher risk in first birth at 25–39 years and last pregnancy at ages 30–39 years. However, 10.03% of abnormal embryonic development, 8.96% of trauma; 5.99% of maternal disease and other 0.68% are stated to cause these reproductive abnormalities
[74]	Examine the relationship between abnormal menstrual cycle length (AMCL) and exposure to petrochemicals in a population of petrochemical workers in Beijing China	Beijing, China	Quantitative Cross-sectional retrospective study Standardized questionnaires	3343 (20–44 years old)	Petro-Chemical	Physical: Hard physical work Organisational: Rotating shift Psychological: Stress Ergonomic: long hours of standing Chemical: Benzene, dust, gasoline, manganese, acid, lime dust, hydrogen sulphide, ammonia, and toluene	Prenatal: Menstruation and cycle disorder, Hormonal imbalance	AMCL cases among 10% of the female workers (7% with long cycle and 98% had a short cycle). Main causes: obesity, activity of the victims in the Oil refinery, exposure to benzene. Stress and the AMCL cause hormone imbalance
[75]	Investigate the association between birth weight and exposure to benzene, work stress, other occupational and environmental hazards with adjustment for gestational age	Beijing, China	Quantitative Air sampling, chemical measurements, workers classification (standardized algorithm, toxicology check list, birth weight assessment	792 (20–40 years old)	Petro-Chemical	Chemical: Benzene Psychological: work stress	Postnatal: Birth weight	Low exposure to benzene leads to reduced newborn babies’ weight and work stress. Further, the interrelationship between benzene, work stress and other occupational hazards deducts 183g of birth weight compared to those unexposed
[76]	Examine whether an association exists between low-level exposure to organic solvents and menstrual patterns in women employed in a large petrochemical industry in Beijing, China	China	Quantitative Cross sectional	1408 (Age not given)	Petro-Chemical	Chemical: Organic/aromatic solvents (benzene, styrene, toluene, or xylene)	Prenatal: Menstruation and cycle disorder	Aromatic solvents increase the frequency of oligomenorrhea. Increase to 7% for each year of exposure. The job’s difference, degree of exposure and presence of other chemicals and affect the menstruation and cycle. The effect of additional chemicals is low compared to volatile organic solvents, which is apparent after 3 years of exposure
[77]	Investigate the association of birth weight with maternal and paternal exposure to organic solvents	Beijing, China	Quantitative Exposure assessment (questionnaire)	1222 (Optimal reproductive age)	Petro-Chemical	Chemical: exposure to low concentration of benzene, toluene, xylene, and styrene	Postnatal: Birth weight	Maternal exposure to benzene reduces newborn weight at both individual and parental exposure. Any small increase of benzene concentration in the atmosphere raises the gravity of the impacts. This issue is more significant among female babies and younger mothers’ babies
[78]	Investigate associations between estimated maternal occupational oil mist exposure during pregnancy and birth defects using population-based case-control data	United States	Quantitative Case control study Retrospective exposure assessment	30151 (>20 years old)	Manu-Facturing/Transport	Chemical: Occupational Oil mists	Postnatal: Birth defects	Septal heart defects observed in female labour exposed to oil mists during one-month preconception until the third month of pregnancy (212.5 μg/m^3^-h). 5 out of 8 CHD phenotypic groups showed elevated odds ratio while only one of 10 non-heart defects displayed an elevated odds ratio. The exposure to mineral oil products leads to the development of congenital heart defect

### Study Characteristics

This review has utilised 15 published articles and one unpublished source. Eight of the 16 studies used a quantitative research method with sample sizes ranging from 792 to 3,343 for the petrochemical operations, and 1,035 to 44,000 for the mining [71–78]. Three studies applied qualitative research methods, often combined with a literature review and empirical investigation, with all participants coming from mining [79–81]. Two other studies in mining applied a mixed methods with 156–686 respondents [72, 81–83]. Two other studies were transaction and conference papers which examine the OHS in the mining industry in South Africa [84, 85]. Transaction paper refers to a paper that meets the requirements of a conference paper. These two articles are constituted of general information without clear description of the methods. However, both met the inclusion criteria of this scoping review and fostered a balanced picture of available evidence relating to the scope of the review. Studies were conducted in both developed and developing countries, such as South Africa [71, 79–82, 84–86]; Australia [80, 83]; China [74–77] Indonesia [72] and the United States [78]. Those in South Africa, Australia, and Indonesia focused on mining, whereas those in China were based on the petrochemical industry. The studies in South Africa were related to mining activities [71, 79, 81, 82, 84–86]. The one study in the United States investigated the risks of female SRH by exposure to hydrocarbon oil mists in manufacturing and transport activities [78].

Eleven studies focused on the mining industry, which generally involved work underground and constituted of exploration, quantification, extraction, and processing of different minerals [71–73, 79–86]. These included, iron, ore, coal, gold, copper, aluminium, nickel, zinc, platinum, gold, hard rock, phosphate [71–73, 79–86]. Four studies on petrochemical focused on plants operating, processing and refinery [74–77]. One study investigated exposure to occupational oil mists from manufacturing and transport in the United States [78].

### Occupational Factors of Sexual and Reproductive Health in Oil, Gas, and Mining Industry

**FIGURE 2:**
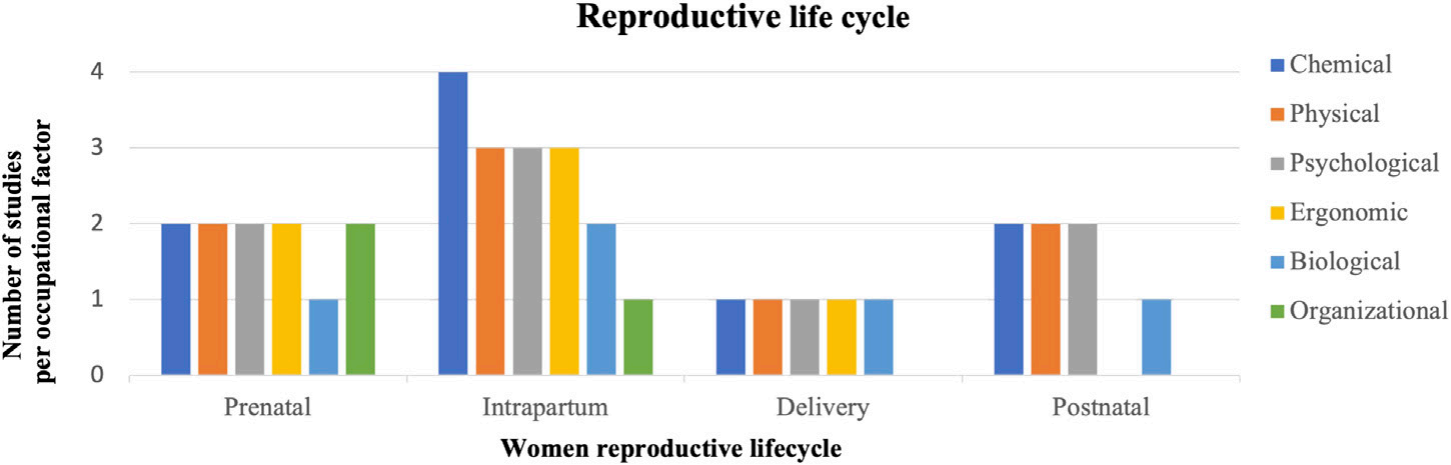
Predominant occupational factors in women’s reproductive life cycle (Queensland, Australia. 2020).

The thematic analysis identified six occupational factors, which potentially affected sexual and reproductive health status among female workers in OGM industry. These factors were: biological, chemical, ergonomic, physical, psychological, and organisational (Figure 2). Within women’s reproductive lifecycle period, precisely for pre to postnatal, chemical hazards posed the most significant threat to sexual and reproductive health, particularly during the intrapartum phase (Figure 2). However, ergonomic, physical, and psychological hazards were also harmful during the intrapartum and prenatal stages.

### Sexual and Reproductive Disorders in Oil, Gas, and Mining Industry

Based on the 16 included studies, Table 3 shows the sexual and reproductive health characteristics experienced by women workers in OGM industry, ranging from infertility to sexual violence.

**TABLE 3:** Sexual and Reproductive Health Characteristics in Oil, Gas and Mining industry (Queensland, Australia. 2020).

Industries	Sexual and reproductive issues
Mining	• Infertility/ subfertility/ effects of motherhood
	• Menstruation and cycle disorder; hormone imbalance
	• Fetal anomalies; congenital malformation
	• Miscarriage (before 28 weeks), stillbirth (after 28 weeks)
	• Risky pregnancy
	• Premature labour
	• Child physical, mental, and developmental disorders
	• Birth weight, birth defects
	• Sexual harassment
	• HIV/AIDS
Petrochemical	• Birth weight
	• Menstruation and cycle disorder; hormone imbalance

Female workers in OGM industry experienced poor sexual and reproductive health outcomes during either prenatal or intrapartum stages [68, 70-73, 76, 78-79, 81] compared with delivery and postnatal periods [71, 76, 80, 87] (Supplementary Appendix S3). Chemical and physical hazards were the most dangerous occupational factors affecting sexual and reproductive health outcomes among female workers in OGM industry (Supplementary Appendix S3) [71, 74–79, 84, 85]. The geographic analysis of the 16 studies revealed a concentration of research in South Africa for the mining industry and in China for the petrochemical industry. Two studies discussed sexually motivated physical and psychological harassment or intimidation, generally linked to organisational factors, which promoted gender discrimination and male dominance [79, 85]. Four studies into African and Indonesian mining reported HIV/AIDS cases among female workers [72, 84–86]. The seroprevalence survey performed in South Africa, Botswana, Zambia disclosed a significant rate of HIV/AIDS among women of different age groups, especially the unskilled and semi-skilled workers (14.6% between 20 and 29 years old, 14.9% between 30 and 39 years and 4.5% between 40 and 49-years [86].

### Key Themes on Occupational Factors and Women’s Sexual and Reproductive Health

#### Biological

Biological factors were the least discussed in the included studies. In fact, only one conference report of South African Institute of Mining and Metallurgy Hard Road Safety in 2009 discussed exposure of female workers in hard rock mining to various biological hazards, such as cytomegalovirus, hepatitis, HIV, rubella, varicella, Toxoplasma gondii [85]. This report highlighted that exposure to these microorganisms was associated with menstruation and cycle disorder, teratogenesis, gene mutation, pregnancy complications, miscarriage, stillbirth, preterm birth, and other development-related abnormalities in foetal and children [85].

#### Organizational

Five studies identified organisational factors as influencing female workers sexual and reproductive health in mining [79, 80, 82, 83]. Most were qualitative and found risky pregnancy and menstruation and cycle disorders as the main sexual and reproductive health concerns [71–73,75, 86]. Some of the organisational factors affecting sexual and reproductive health were FIFO/DIDO system-related schedule and gender discrimination in Australia, racist and discriminative management and policies, and violence in South Africa. For instance, women in South Africa Mining preferred to hide their pregnancy to secure their job [81]. Female black South African miners felt disadvantaged as they were not re-allocation to light duties during pregnancy compared to their white counterparts [79]. In Australia, women of childbearing age complained about their difficulties to have a proper work-life balance due to FIFO/DIDO system and its long, inflexible work hours [83].

#### Chemical

Nine studies showed chemical factors affected female workers’ sexual and reproductive health status [71, 74–79, 84, 85]. Some of the chemical hazards reported included organic and inorganic solvents in the five petrochemical quantitative studies in China and United States [75–78] and heavy metals, toxic gases and dust, pesticide, alcohol, and tobacco smoke in the four studies on South Africa mining studies [71, 79, 84, 85]. For example, Badenhorst and Platinum (2009) report that the exposure to lead can affect women miners’ pregnancy at early stages (8–10 weeks) by crossing the placenta and damaging the foetus [85].

#### Physical

Physical factors associated with sexual and reproductive health among female workers in OGM industry was also identified in eight of the papers [71, 73, 74, 79, 81, 82, 84, 85]. Menstruation and cycle disorders and risky pregnancy were recognised to be triggered by physical factors. For example, a study by Calitz (2004) found South African female miners suffer from painful menstrual periods due to their exposure to the underground mining hot or cold environments [79]. Six studies in South Africa pointed out the different physical hazards involving radiation, heat, noise and vibration, mechanical shocks, physical strain, male-designed tools and equipment [71, 79, 82, 84, 85]. While the physical factors influencing menstruation disorders and miscarriage in the petrochemical industry were physical exertion and fatigue, caused by strenuous activities, prolonged standing, long working shift, heavy equipment vibration and underground heat stress [74, 75].

#### Ergonomic

Ergonomic factors adversely impacted women’s sexual and reproductive health in five studies, particularly risky pregnancy [74, 79, 82, 85]. Three qualitative research and reports related to South Africa mining suggested that women have difficulties to use the existing equipment, including the working environment, which might affect their sexual and reproductive health status [71, 80, 86]. For example, Zungu (2011) highlights that the constant use of oversized and heavy tools and equipment, manual handling and lifting activities, and other ranges of motions cause stress and fatigue affecting hormone levels, fluid balance, and reducing fertility [71].

#### Psychological

Psychological factors influenced sexual and reproductive health in six studies [71, 73–75, 79, 85]. Menstrual disorders and risky pregnancy were the main consequences of workplace stress, anxiety, and depression. Studies from South Africa detailed male harassment, gender discrimination, and work-life imbalance, which led to stress, frustration, and pressure among female workers [71, 73, 79, 85]. In contrast, the quantitative studies in China’s petrochemical operation found workplace stress as the outcomes of strenuous physical activities, long hours of standing, and an inflexible work schedule [74, 75].

## Discussion

This scoping review demonstrates the scarcity of global research on the influence of occupational factors on female workers’ sexual and reproductive health outcomes in OGM industry. Nevertheless, the geographic analysis suggests a concentration of the research mainly in low-and middle-income countries. This could be aligned with the amendment of policies and legislation promoting women’s employment and gender equality in South Africa mining [87] and the significant economic profits generated by the petrochemical industry in China [88, 89]. Despite being limited, the findings show that chemical and physical hazards could be considered as potential risk factors for sexual and reproductive health disorders among women workers in OGM industry. The included studies suggest menstrual and cycle disorders due to chemical hazards, such as heavy metals, toxic gases, and dust. Similar findings were also reported in other industries, such as plastic, agriculture, health care, waste management and construction where chemical hazards are detrimental factors to women’s sexual and reproduction system [90–94]. Hence, this study adds to the growing body of evidence of the detrimental impact of chemic hazards on women’s sexual and reproduction system.

Menstruation and cycle disorders were also negatively affected by physical factors, such as heat, noise, dust, and physically demanding work activities also placed pregnancy at risk [71, 74, 79, 82, 85]. This is in line with literature, which suggests that strenuous exercise during pregnancy can have negative physiological outcomes, such as hormonal imbalance, vasoconstriction, myometrial contraction, reduced plasma volume, and diversion of blood flow away from the placental bed causing foetal hypoxia [95]. In addition, there is evidence to suggest that poor work organisation can adversely affect menstruation and pregnancy in OGM industry [74, 79, 81, 82, 85]. For example, an occupational health-related study indicates that shift work and irregular work schedules in different industries can change women’s circadian rhythm causing menstrual irregularities and pregnancy complications, such as gestational hypertension, preterm delivery, small for gestational age baby and preeclampsia [95]. This has been observed in a few women working in petrochemical engineering, medical industry, and metallurgy with a high proportion of abnormal periods, reproductive system infection and infertility [96].

Psychological factors are also reported to be associated with menstruation and cycle disorders, including stress, anxiety, constant feeling of fear and pressure due to the strenuous physical work and male counterparts’ harassment [71, 73–75, 79, 85]. This is concerning as OGM activities are recognised as stressful in both developed and developing countries; however, no studies clearly investigate its association with female sexual and reproductive health status [97–99]. Existing studies in the mining industry were based on reports or qualitative self-reported methods that rely on the participants’ experience and their perceptions on the psychological factors relating to sexual and reproductive health issues [71–73, 79–86]. In contrast, those in the petrochemical industry applied quantitative measures and analysis [74–77]. This disregards women workers’ opinions and experience of their working conditions and the health and safety outcomes in the petrochemical industry. Besides, it excludes other types of occupational hazards that can affect their SRH.

Existing studies on organisational factors of sexual and reproductive health disorders in OGM industry mostly used qualitative methods or reports in which female workers’ perceptions and experiences in underground mining are explored [74, 79–83, 85]. However, research suggests that employers’ leadership and duties towards employees’ health and wellbeing are the fundamental keys that need to be investigated through legislation, policies, organisation culture and management systems [100]. Laws, regulations, and organisational health and safety management systems are powerful tools to influence safety leadership, preventive culture, and workers’ positive behaviour and commitment that mitigate the risks of exposure to workplace hazards [101–105].

Concerning the physical factors, this scoping review found that body stress, heavy tools, illumination, heat, heights, noise, vibration, electromagnetic radiation, ventilation pressure potentially harm female workers sexual and reproductive health in the mining industry [71, 74, 79, 82, 85]. These physical hazards are perceived as less harmful than chemical hazards in oil and gas industry and have been underrated because of their chronic effects on individual health [106]. Hence, workers are largely unaware of these hazards and the severity of the risks [106]. Different investigations confirmed that physical hazards can cause chronic diseases and mortality, involving sexual and reproductive health issues [106–110]. This is due to fatigue, stress, pain, body mechanism disorders and damage of the internal organs and tissues [106–110].

The findings of this scoping review suggest a hostile working environment for women, including organisational structure, management systems, practices, and facilities in OGM industry [71,74,79,85]. For example, the male-designed ablution facilities, change houses and bathrooms without privacy, ill-fitting personal protective clothing, machinery and equipment, heavy tools, social environment of male hostility, violence, and sexual assaults [71, 74, 75, 79, 85]. These circumstances subsequently affect women’s psychological state due to the lack of comfortability and the stress, anxiety, or frustration they experience. The selected studies reveal the development of stress and anxiety as a result of organisational and physical factors involving strenuous work, gender discrimination, work-life balance, and harassment [71, 73–75, 79, 80]. Other studies also highlight different issues affecting women’s psychology in OGM industry, including professional and social isolation, loneliness, women’s identity on masculinity, and uncompromising supervisors [111–113]. Hence, further research is needed to investigate industries’ management and organisational system, including policies, programs, and implementation, to address such circumstances and recommend implementable strategies that promote women’s health and wellbeing.

Finally, a few cases of HIV/AIDS among female workers in OGM industry have been reported in several included studies [84–86]. Men were claimed to be the source of transmission [85]. However, this claim requires further investigation and empirical evidence as this statement is obtained from a report with limited scientific study. Studies also raise the issues of underreported HIV/AIDS cases at the workplace to prevent stigma [114–116].

### Implications for Future Research

The findings of this scoping review have several implications for future research. Firstly, future research should consider quantitative research methods to obtain high quality evidence of the relationship between occupational hazards and female workers’ sexual and reproductive health. Longitudinal research designs to better establish the causal relationship between exposure to occupational hazards and female workers’ sexual and reproductive health would be valuable. Additionally, new studies should consider the analysis of legislation, policies and programs related to gender diversity and women’s health in developed and developing countries, and across OGM industry to help understand its effects on the organisational culture and management of female sexual and reproductive health status. Also, future studies should identify all potential biological, chemical, and environmental health hazards, particularly air and water pollutants, which are often found in OGM industry. Environmental health hazards including heavy metals, carbon monoxide, nitrogen oxide, sulfur dioxide and particulate matter can be detrimental to women’s reproductive system [105, 117–122]. Yet, this scoping review has shown that there is very little research and knowledge on these hazards. The consideration of women’s differences in biological, physiological characteristics, and physical capacities in research and workplace risk assessment is also needed and justified by other researchers [123,124]. This is crucial for a specific and effective control of health and safety concerns of women in OGM industry. The combination of the epidemiologic studies, ergonomic assessment checklist tools and medical examination is needed to ensure the accuracy of the investigation [19]. From the psychological factor perspectives, future studies should consider occupational mental health and wellbeing, particularly in the developing countries where mental health issues are still perceived as Western luxury or religious-based punishment [125]. Finally, future research should include HIV/AIDS and sexual health in OGM industry to investigate the source and magnitude of the risks and develop preventive and reactive strategies and programs.

The findings also have managerial, policy and practical implications. In addition to the potential of exposure to multiple hazards in OGM industry, there are specific health issues that manifest exclusively in women requiring thorough investigation and control measures. Moreover, women in OGM industry are reported to endure various forms of gender inequality, including discriminatory policies and practices, violence, sexual harassment due to the persistent traditional male norms. These unfair situations still occur in the workplace because women’s employment concerns remain overlooked in the industry’s investigations [126]. Considering the economic benefits of increasing women’s workforce participation in the industries, promoting a female friendly workplace appears to be crucial [127]. It consists of developing organisational and managerial policies and practices that respond to women’s special needs. Studies should therefore be conducted to investigate the current policy making, organisational structures, management systems and practices in OGM industry and identify their suitability to women’s health, safety, and wellbeing. Some of the specific strategies to support women may include policies, hazards identification, risk assessments control strategies and interventions which pose a risk to their health and wellbeing. Human resources management systems, including recruitment processes, resources provision, development opportunities, and organisation’s policies and practices that support work-life balance for female employees could also be implemented [127–131]. However, future research is needed to more coherently identify which of these strategies women may find most beneficial to their career advancement.

### Limitations

This scoping review has few limitations. Firstly, it did not cover gynaecological and breast cancers, which are also part of women’s sexual and reproductive health issues Secondly, since there was no evaluation of the quality of the included studies in this scoping review, the accuracy and validity of the findings remain controversial and require thorough scientific research. Nevertheless, all expected occupational factors relating to women’s SRH were identified in the relevant studies. Additionally, because our review was restricted to English language articles, we may have missed key articles in non-English. This scoping review also exhibited heterogenous SRH characteristics among female workers in OGM industry.

### Conclusion and Future Research

Research on occupational factors influencing sexual and reproductive health status among women workers in OGM industry is scarce and limited. Nevertheless, the included studies display various sexual and reproductive health issues throughout women reproductive lifecycle periods (prenatal, intrapartum, delivery, and postnatal) as the results of the exposure of OGM occupational hazards. Chemical and physical hazards were found to have substantial effects on women workers SRH in OGM industry, while menstrual and cycle disorders and risky pregnancy are the major SRH health concerns. Recognising the lack of quality assessment of the included studies, the accuracy and the validity of the findings and interpretation are constrained. However, this review is a preliminary step to inform about the key characteristics and occupational factors of SRH in OGM industry, as mapped in the literature. Empirical studies are recommended for an evidence-based decision making, initiatives, policies, and practices promoting a gender-based human resources and health and safety management systems in OGM industry.
